# Study on Four-Wave Mixing Enhancement in Nanofiber Systems

**DOI:** 10.3390/nano16211410

**Published:** 2026-11-02

**Authors:** Yilin Zhu, Chaohua Tan

**Affiliations:** 1 School of Engineering, University of Southampton, Southampton SO17 1BJ, UK; 2 School of Physics and Electronics, Shandong Normal University, Jinan 250014, China

**Keywords:** four-wave mixing, nanofiber, nonlinear optics, electromagnetically induced transparency, spatial mode overlap, quantum optics, mean-field approximation

## Abstract

We investigate four-wave mixing (FWM) in a five-level atomic system coupled to an optical nanofiber. The transverse inhomogeneity of the guided field is incorporated through a mode-weighted mean-field approximation, yielding effective one-dimensional propagation equations. The conversion efficiency is evaluated over different detuning parameters and compared with a uniform-field reference under consistent normalization. For the parameters studied, the nanofiber model predicts higher peak conversion efficiencies, accompanied by distinct changes in the spectral response. The two-photon resonance narrows, the upper-transition resonance retains nearly the same bandwidth, and the range of probe detunings supporting appreciable conversion broadens. To account for the interplay among detuning parameters, particle swarm optimization (PSO) is employed, yielding a best-found efficiency of $1.85\times 10^{-3}$ within the prescribed search bounds. The resulting two-dimensional efficiency map exhibits a tilted high-efficiency band, revealing a correlated dependence on the detunings rather than independently optimized resonances. These findings clarify how transverse mode confinement and coupled detuning conditions shape the spectral response of FWM in the nanofiber–atom system.

## 1. Introduction

Four-wave mixing (FWM) is a third-order nonlinear optical process that enables frequency conversion, phase conjugation, and coherent amplification [1,2]. In multilevel atomic systems, coherent light–matter interactions can strongly reshape the nonlinear response. The process is commonly described through the third-order nonlinear susceptibility $\chi^{\left(3\right)}$, while the generated frequency is constrained by energy conservation [1,3,4]. Coherent-control mechanisms such as electromagnetically induced transparency (EIT) and Raman resonance can reduce resonant absorption and modify nonlinear dispersion, thereby enabling FWM at low optical intensities [5,6,7]. Experimental and theoretical studies of double-Lambda and four-level systems have further demonstrated strong control of the conversion response through detunings and coupling-field strengths [5,8,9]. In the five-level EIT-FWM scheme of Wu et al. [3], suppression of multiphoton absorption was proposed as a route to large conversion enhancement; however, the interpretation of that enhancement was subsequently debated in a Comment and Reply [10,11]. In this work, we distinguish between the effects of EIT-induced absorption suppression and nanofiber mode confinement on the calculated FWM response. Beyond coherent control, optical confinement in waveguides and micro/nanofibers provides an additional route to strengthen light–matter interactions through reduced effective mode areas and enhanced local fields [12,13]. As integrated and micro/nanophotonics continue to develop, optical platforms are increasingly moving toward miniaturization, integration, and low-power operation [14]. In free-space geometries, comparatively large optical mode areas limit the field amplitude attainable for a given optical power, whereas waveguides and other micro/nanoscale structures can concentrate optical energy into subwavelength transverse dimensions [15,16,17,18]. This confinement can increase local field amplitudes and nonlinear polarization, making guided photonic systems attractive for compact nonlinear optical devices [19]. Optical micro/nanofibers are particularly useful because they combine extended propagation lengths with an evanescent field accessible to surrounding atoms [20,21]. Their guided modes are strongly inhomogeneous in the transverse plane [22,23,24,25], so the nonlinear response depends on both field amplitude and spatial mode structure. Consequently, a spatially uniform plane-wave treatment cannot capture all position-dependent contributions, and an effective model should retain the guided-mode profile or the associated mode-overlap integrals. Recent work on structured-light FWM has likewise emphasized the role of spatial-mode overlap in determining the generated field [26]. For subwavelength nanofibers, a substantial fraction of the guided-mode energy extends outside the dielectric surface, enabling direct interaction with nearby atoms while retaining a small effective mode area [21,27,28]. To investigate the influence of transverse guided-mode inhomogeneity on EIT-assisted four-wave mixing (FWM), we consider a five-level atomic system coupled to the fundamental HE_11_ mode of an optical nanofiber. The spatial distribution of the guided field is accounted for through a guided-mode-weighted mean-field approximation, yielding an effective one-dimensional propagation model. We calculate the FWM conversion efficiency over a range of detuning parameters and compare the resulting spectral response with that of a uniform-field reference under the same normalization. The calculated spectra exhibit different responses to the individual detunings. Compared with the uniform-field case, the resonance associated with $\Delta_{3}$ becomes narrower, while the bandwidth associated with $\Delta_{43}$ shows little change. By contrast, the detuning range over which appreciable conversion occurs becomes broader for $\Delta_{2}$. We also employ particle swarm optimization (PSO) to explore the joint dependence of the conversion efficiency on the detuning parameters. The two-dimensional efficiency map exhibits a tilted high-efficiency band, indicating that favorable operating conditions are associated with correlated detunings rather than independently optimized resonances. These results highlight the distinct spectral responses to different detuning parameters and the importance of their combined variation in determining the conversion characteristics of the nanofiber-coupled atomic system. The model is intended as a proof-of-principle description; quantitative comparison with warm-vapor experiments requires additional effects, including Doppler averaging, transit-time broadening, atom–surface shifts, and an experimentally specified mode normalization [21,29,30].

## 2. Theoretical Model

**Figure 1:**
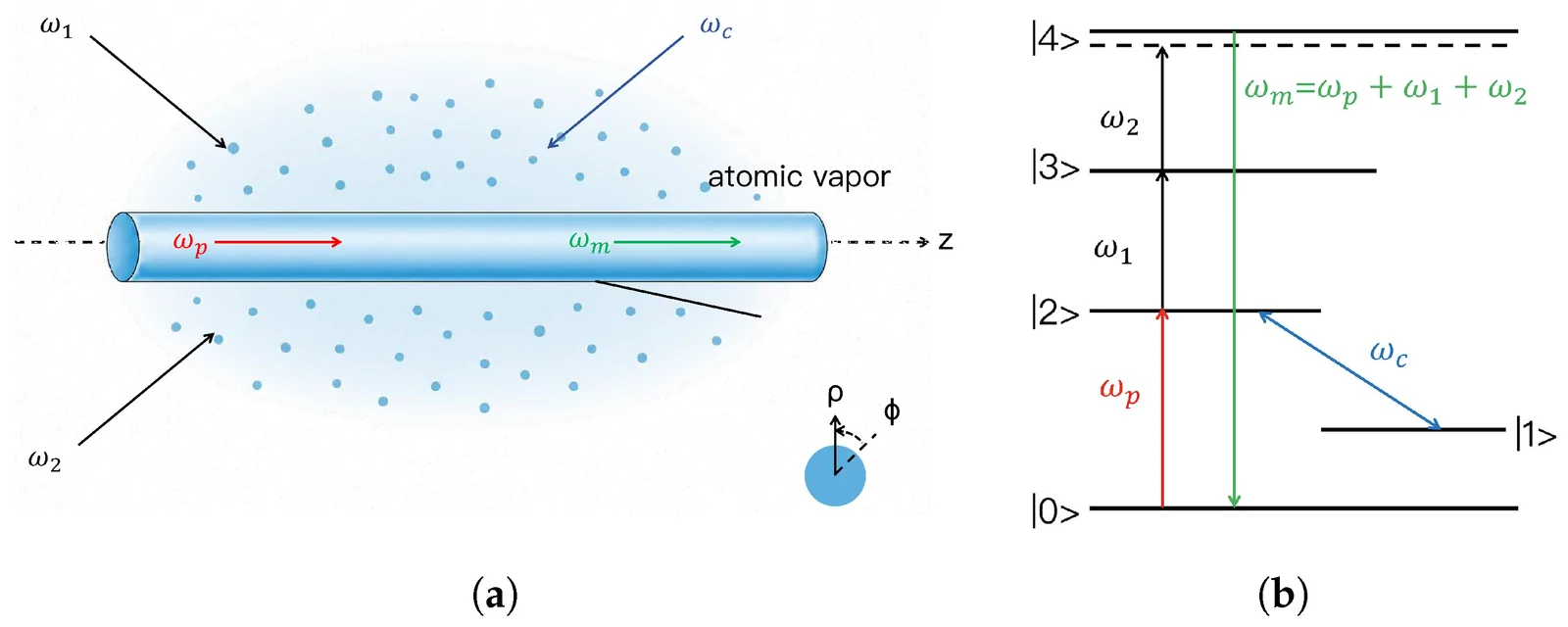
(**a**) Schematic of the nanofiber-coupled four-wave mixing (FWM) system. The probe field $\omega_{p}$ and the generated FWM field $\omega_{m}$ propagate along the nanofiber in the fundamental guided mode ${\mathrm{HE}}_{11}$, while the control field $\omega_{c}$ and the driving fields $\omega_{1}$ and $\omega_{2}$ interact with the surrounding atomic medium as external fields. The cylindrical coordinates (r,ϕ,z) are indicated. (**b**) Corresponding five-level energy-level diagram of the FWM process. The probe field $\omega_{p}$ drives the transition |0〉→|2〉, the control field $\omega_{c}$ couples |1〉↔|2〉, and the fields $\omega_{1}$ and $\omega_{2}$ drive the transitions |2〉→|3〉 and |3〉→|4〉, respectively. The generated field satisfies the energy-conservation relation $\omega_{m}=\omega_{p}+\omega_{1}+\omega_{2}$.

We consider an optical nanofiber interacting with surrounding rubidium vapor, as illustrated in Figure 1a. In this structure, the probe field $\Omega_{p}$ is launched into the nanofiber and propagates as the fundamental mode ($HE_{11}$ mode) [31]. In this system, the fiber diameter is much smaller than the operating wavelength, so the propagating optical field produces a pronounced evanescent field at the fiber surface [15,28], enabling effective light-atom interaction with the surrounding atomic medium. The generated FWM field ($\Omega_{m}$) is also assumed to be coupled into the guided mode and propagates along the nanofiber. In contrast, the control field ($\Omega_{c}$) and the two driving fields ($\Omega_{1}$) and ($\Omega_{2}$) are treated as external plane-wave incident on the atomic medium. Because of the small mode volume and strong spatial confinement of the nanofiber, the optical field exhibits a significantly enhanced electric-field distribution in the vicinity of the fiber surface [15,31]. More importantly, the evanescent guided field is strongly nonuniform in the transverse direction, resulting in spatially dependent Rabi frequencies and nonlinear coupling strengths. Compared with conventional free-space systems, the confined guided field provides stronger local coupling near the nanofiber surface and introduces a position-dependent nonlinear response, both of which can modify the FWM conversion characteristics [1,15].

We assume the five-level FWM scheme shown in Figure 1b, which is designed to suppress resonant absorption via the EIT effect while simultaneously realizing efficient FWM. The levels are defined as follows. State |0〉 is the atomic ground state and serves as the initial population state of all transitions in the system. State |1〉 is a metastable state which, together with |0〉, forms a long-lived spin coherence [32,33]. The intermediate excited states |2〉 and |3〉 are responsible for coupling to the external optical fields, and |4〉 is a higher excited state. Under optical driving, a probe field excites the atoms from the ground state |0〉 to the intermediate state |2〉. Meanwhile, a coupling field acts on the |1〉↔|2〉 transition, suppressing the resonant absorption of the probe on the |0〉↔|2〉 transition through EIT and preparing the atoms in a coherent dark state. Subsequently, the driving field $\omega_{1}$ couples the transition |2〉→|3〉, while $\omega_{2}$ couples the transition |3〉→|4〉. The nonlinear polarization associated with the |4〉↔|0〉 transition acts as the source of the generated FWM field. This process satisfies the energy-conservation relation $\omega_{m}=\omega_{1}+\omega_{2}+\omega_{p}$ and the phase-matching condition $k_{m}=k_{1}+k_{2}+k_{p}$ [1], where $\omega_{1}$ and $\omega_{2}$ denote the two driving fields, $\omega_{p}$ denotes the probe field, and $\omega_{m}$ denotes the newly generated field arising from the FWM process [1]. The five-level scheme shown in Figure 1b may be realized using selected hyperfine states of Rb87. One possible assignment is $|0\rangle =5S_{1/2}\left(F=1\right)$, $|1\rangle =5S_{1/2}\left(F=2\right)$, and $|2\rangle =5P_{3/2}\left(F^{\prime }=2\right)$, with the probe and control transitions near 780nm. The remaining states may be identified as $|3\rangle =6S_{1/2}\left(F=1\right)$ and $|4\rangle =6P_{3/2}\left(F^{\prime }=2\right)$. The transitions |2〉↔|3〉 and |3〉↔|4〉 then correspond to wavelengths of approximately 1367nm and 2732nm, respectively, while the generated field is associated with the |4〉→|0〉 transition near 420nm. This assignment provides a possible atomic realization of the proposed level structure, subject to appropriate choices of polarization and magnetic sublevels. The present calculations, however, are based on an effective five-level model rather than a hyperfine-resolved treatment of Rb87. A quantitative description of this atomic implementation would require transition-specific dipole matrix elements, decay rates and branching ratios, together with the wavelength-dependent guided-mode profiles and phase-matching conditions.

Within the semiclassical theory, the optical fields are treated as classical electromagnetic fields. The *j*-th optical field in the system can be written as $E_{j}\left(z,t\right)=\frac{1}{2}\left(E_{j}e^{i(k_{j}z-\omega_{j}t)}+c.c.\right)$, where $E_{j}$ is the complex amplitude, $k_{j}$ is the wave vector, and $\omega_{j}$ is the optical frequency. In the nanofiber system, due to guided-mode confinement, the optical field possesses a transverse intensity distribution. In the present model, the electric-field amplitudes of $\Omega_{p}$ and $\Omega_{m}$ can be written as $E_{j}\left(r\right)=E_{j}u_{j}\left(r,\phi \right)$, where $u_{j}\left(r,\phi \right)$ is the transverse distribution function of the $HE_{11}$ mode. By contrast, $\Omega_{c}$, $\Omega_{1}$, and $\Omega_{2}$ are treated as external broad fields, whose transverse variation is neglected in the effective interaction region. The half-Rabi frequency for each optical transition is defined as $\Omega_{j}=\mu_{ij}E_{j}/2\hbar$ (j=1,2,p,m,c), where $\mu_{ij}$ represents the electric-dipole matrix element for atomic transition between state |i〉 and |j〉. Therefore, we have $\Omega_{p}=\frac{\mu_{02}E_{p}}{2\hbar }$, $\Omega_{1}=\frac{\mu_{23}E_{1}}{2\hbar }$, $\Omega_{2}=\frac{\mu_{34}E_{2}}{2\hbar }$, $\Omega_{c}=\frac{\mu_{12}E_{c}}{2\hbar }$, and $\Omega_{m}=\frac{\mu_{04}E_{m}}{2\hbar }$. Since the probe field $\Omega_{p}$ and the generated field $\Omega_{m}$ are guided modes, their local Rabi frequencies are $\Omega_{p}\left(r,\phi \right)=\Omega_{p}^{0}\zeta_{p}\left(r,\phi \right),\;\Omega_{m}\left(r,\phi \right)=\Omega_{m}^{0}\zeta_{m}\left(r,\phi \right)$, where $\Omega_{p}^{0}$ and $\Omega_{m}^{0}$ are the reference Rabi frequencies evaluated at the field maximum of the guided mode. Therefore, the nonlinear interaction experienced by atoms is different at different transverse positions, and the total FWM response should be obtained by integrating the local contribution over the guided-mode distribution. Where explicitly assumed, the probe and generated fields are approximated by a common normalized transverse profile, $\zeta_{p}$ and $\zeta_{m}$ are denoted collectively as ζ.

Under the electric-dipole approximation and the rotating-wave approximation (RWA), the interaction process between multiple coherent optical fields and a multilevel atomic system is described by the following effective Hamiltonian [32]:

(1) $${\hat{H}}_{int}=-\hbar \left(\sum_{j=1}^{4}\Delta_{j}\left|j\rangle \langle j\right|+\zeta \Omega_{p}\left|2\rangle \langle 0\right|+\Omega_{1}\left|3\rangle \langle 2\right|+\Omega_{c}\left|2\rangle \langle 1\right|+\Omega_{2}\left|4\rangle \langle 3\right|+\zeta \Omega_{m}\left|4\rangle \langle 0\right|\right)+h.c.,$$

where ζ denotes the normalized transverse mode function of the nanofiber-guided field. To eliminate the time dependence in the Hamiltonian, a rotating frame is introduced, and the field detunings are defined as $\Delta_{1}=\omega_{p}-\omega_{c}-\omega_{10}$, $\Delta_{2}=\omega_{p}-\omega_{20}$, $\Delta_{3}=\omega_{p}+\omega_{1}-\omega_{30}$, $\Delta_{4}=\omega_{p}+\omega_{1}+\omega_{2}-\omega_{40}$, and $\Delta_{43}=\Delta_{4}-\Delta_{3}$, where $\omega_{j0}=\left(E_{j}-E_{0}\right)/\hbar$ represents the intrinsic atomic transition frequency from ground state |0〉 to excited state |j〉, with $E_{j}$ being the eigenenergy of atomic state |j〉. Since the rapid time oscillations of the light field are eliminated through the rotating-wave approximation, and the spatial oscillations caused by the mismatch of wave vectors are eliminated through the phase-matching condition, in the subsequent derivation, it will be assumed that the phase-matching condition has been satisfied, and the residual wave-vector mismatch terms will no longer be retained.

The probe field and the generated four-wave mixed signal light are the guided modes of the nanofiber, while the control field and the driving field are treated as external plane-wave fields. Based on this, each electric field can be expressed as:

(2a)Ep=Ep(z,t)up(r,ϕ)ei(kpz−ωpt)+c.c.,(2b)Em=Em(z,t)um(r,ϕ)ei(kmz−ωmt)+c.c.,(2c)Ec=Ecei(kcz−ωct)ec+c.c.,(2d)E1=E1ei(k1z−ω1t)e1+c.c.,(2e)E2=E2ei(k2z−ω2t)e2+c.c.

where $u_{j}\left(r,\phi \right)$ is the normalized transverse mode-distribution function of the nanofiber-guided field and $k_{j}$ is the corresponding propagation constant. The mode-distribution function $\zeta_{j}\left(r,\phi \right)=\sqrt{{\left|e_{j,r}\right|}^{2}+{\left|e_{j,\phi }\right|}^{2}+{\left|e_{j,z}\right|}^{2}}$ is defined as the scalar amplitude of the electric field. In the numerical calculations, it is normalized such that max(ζ)=1.

By substituting the Hamiltonian in Equation (1) into the Schrödinger equation, the probability amplitudes $A_{j}$ obey:

(3a)i∂∂t+d0A0+ζ∗Ωp∗A2+ζ∗Ωm∗A4=0,(3b)i∂∂t+d1A1+Ωc∗A2=0,(3c)i∂∂t+d2A2+ΩcA1+ζΩpA0+Ω1∗A3=0,(3d)i∂∂t+d3A3+Ω1A2+Ω2∗A4=0,(3e)i∂∂t+d4A4+ζΩmA0+Ω2A3=0

with the complex detuning parameters $d_{j}=\Delta_{j}+i\gamma_{j}$ ($d_{0}=0$, j=1,2,3,4), $\sum_{j=0}^{4}{\left|A_{j}\right|}^{2}=1$. Here, $A_{j}$ denotes the time-dependent probability amplitude of atomic state |j〉, and $\gamma_{j}$ represents the transverse relaxation decay rate of the corresponding atomic level |j〉.

Under the EIT condition, $A_{0}≃1$, and we introduce the Fourier transforms:

(4) $$\begin{array}{c} A_{j}\left(t\right)\propto \int \alpha_{j}\left(\omega \right)e^{-i\omega t}d\omega , \end{array}$$

(5) $$\begin{array}{c} \Omega_{p}\left(t\right)=\int {\tilde{\Omega }}_{p}\left(\omega \right)e^{-i\omega t}d\omega ,\;\Omega_{m}\left(t\right)=\int {\tilde{\Omega }}_{m}\left(\omega \right)e^{-i\omega t}d\omega \end{array}$$

where $\alpha_{j}\left(\omega \right)$ is the frequency-domain probability amplitude of atomic state |j〉, and ${\tilde{\Omega }}_{p}\left(\omega \right)$, ${\tilde{\Omega }}_{m}\left(\omega \right)$ are the frequency-domain Rabi frequencies of the probe and FWM signal fields, respectively. The propagation of the probe field $\Omega_{p}$ and the FWM field $\Omega_{m}$ is governed by Maxwell equations. Along the nanofiber axis *z*, we adopt the one-dimensional propagation approximation. Under the EIT condition, $A_{0}≃1$, and the field equations can be written as:

(6a)i∂∂z+1c∂∂tζΩ˜p+κ02α2α0=0,(6b)i∂∂z+1c∂∂tζΩ˜m+κ04α4α0=0

where $\alpha_{0}=1$, $\kappa_{02}=\frac{2N\omega_{p}{\left|D_{02}\right|}^{2}}{c\hbar },\;\kappa_{04}=\frac{2N\omega_{m}{\left|D_{04}\right|}^{2}}{c\hbar }$ and *N* is the atomic density. $D_{02}$ and $D_{04}$ are the electric dipole matrix elements of the transitions, respectively. *c* is the speed of light in vacuum. By Fourier transforming the amplitude equations, one obtains the following algebraic equations in the frequency domain:

(7a)(ω+d1)α1+Ωc∗α2=0,(7b)(ω+d2)α2+Ωcα1+Ω1∗α3=−ζΩ˜p,(7c)(ω+d3)α3+Ω1α2+Ω2∗α4=0,(7d)(ω+d4)α4+Ω2α3=−ζΩ˜m,

We define $A=\left(\omega +d_{1}\right)\left(\omega +d_{2}\right)-{\left|\Omega_{c}\right|}^{2}$ and $B=\left(\omega +d_{3}\right)\left(\omega +d_{4}\right)-{\left|\Omega_{2}\right|}^{2}$. Solving these equations yields the atomic probability amplitudes:

(8) $$\begin{array}{cc} \; & \alpha_{2}=-\zeta \left[\frac{(\omega +d_{1})B}{S}{\tilde{\Omega }}_{p}+\frac{\left(\omega +d_{1}\right)\Omega_{1}^{*}\Omega_{2}^{*}}{S}{\tilde{\Omega }}_{m}\right], \end{array}$$

(9) $$\begin{array}{cc} \; & \alpha_{4}=-\zeta \left[\frac{\left(\omega +d_{1}\right)\Omega_{1}\Omega_{2}}{S}{\tilde{\Omega }}_{p}+\frac{\left(\omega +d_{3}\right)A-\left(\omega +d_{1}\right){\left|\Omega_{1}\right|}^{2}}{S}{\tilde{\Omega }}_{m}\right], \end{array}$$

(10) $$\begin{array}{cc} \; & S=AB-{\left|\Omega_{1}\right|}^{2}\left(\omega +d_{1}\right)\left(\omega +d_{4}\right) \end{array}$$

To express the Maxwell equations in a compact coupled-wave form, the atomic response terms are written as:

(11) $$\begin{array}{cc} \; & \kappa_{02}\alpha_{2}=\zeta \left(g_{2}{\tilde{\Omega }}_{m}+f_{2}{\tilde{\Omega }}_{p}\right), \end{array}$$

(12) $$\begin{array}{cc} \; & \kappa_{04}\alpha_{4}=\zeta \left(g_{4}{\tilde{\Omega }}_{p}+f_{4}{\tilde{\Omega }}_{m}\right) \end{array}$$

Here, $f_{2}$, $f_{4}$ represent self-coupling coefficients describing the field self-modulation, while $g_{2}$, $g_{4}$ denote cross-coupling coefficients responsible for energy exchange between the probe field and the FWM signal field. Substituting $\alpha_{2}$ and $\alpha_{4}$ into Equation (6), respectively, the coupled-wave equations can be obtained as

(13) $$\begin{array}{cc} \; & \frac{\partial {\tilde{\Omega }}_{p}}{\partial z}-i\left(\frac{\omega }{c}+f_{2}\right){\tilde{\Omega }}_{p}=ig_{2}{\tilde{\Omega }}_{m}, \end{array}$$

(14) $$\begin{array}{cc} \; & \frac{\partial {\tilde{\Omega }}_{m}}{\partial z}-i\left(\frac{\omega }{c}+f_{4}\right){\tilde{\Omega }}_{m}=ig_{4}{\tilde{\Omega }}_{p}. \end{array}$$

where $b_{p}=\frac{\omega }{c}+f_{2}$, $b_{m}=\frac{\omega }{c}+f_{4}$, $f_{2}=-\kappa_{02}\frac{\left(\omega +d_{1}\right)\left(\omega +d_{3}\right)\left(\omega +d_{4}\right)-\left(\omega +d_{1}\right){\left|\Omega_{2}\right|}^{2}}{S}$, $g_{2}=-\kappa_{02}\frac{\left(\omega +d_{1}\right)\Omega_{1}^{*}\Omega_{2}^{*}}{S}$, $f_{4}=-\kappa_{04}\frac{\left(\omega +d_{3}\right)A-\left(\omega +d_{1}\right){\left|\Omega_{1}\right|}^{2}}{S}$, $g_{4}=-\kappa_{04}\frac{\left(\omega +d_{1}\right)\Omega_{1}\Omega_{2}}{S}$. $b_{p}$ and $b_{m}$ are the effective complex propagation constants for the probe and FWM fields, respectively. The corresponding eigenvalues are then obtained as

(15) $$k_{\pm }=\frac{b_{p}+b_{m}}{2}\pm \sqrt{{\left(\frac{b_{p}-b_{m}}{2}\right)}^{2}+g_{2}g_{4}}.$$

Under the boundary condition ${\tilde{\Omega }}_{m}\left(z=0,\omega \right)=0$, the expression for the generated FWM field can be obtained as

(16) $${\tilde{\Omega }}_{m}\left(z,\omega \right)={\tilde{\Omega }}_{p}\left(0,\omega \right)\frac{g_{4}}{k_{+}-k_{-}}\left(e^{ik_{+}z}-e^{ik_{-}z}\right).$$

The FWM conversion efficiency is defined as the ratio between the number of generated FWM photons and the number of incident probe photons:

(17) $$\eta =\frac{\left(1/\omega_{m}\right)\int P_{m}\;dt}{\left(1/\omega_{p}\right)\int P_{p}\;dt}$$

where $P_{m}=\hbar \omega_{m}A_{eff}\frac{|\Omega_{m}{|}^{2}}{2\gamma_{4}\sigma_{04}}$ is the power of the generated mixing field and $P_{p}$ is the probe-field power. $A_{eff}$ here represents the effective mode area of the nanofiber guided mode, and $\sigma_{04}$ is the transition cross-section for |0〉↔|4〉.

Because the guided probe and generated fields have nonuniform transverse profiles, the local field response cannot be directly described by a single spatially uniform coupling coefficient. Therefore, an effective one-dimensional description is obtained by introducing a guided-mode-weighted mean-field average over the transverse cross section. This is done by introducing a guided-mode-weighted average over the transverse cross section:

(18) $$\langle f\rangle =\frac{{\;\int \int \;|\zeta \left(r,\phi \right)|}^{2}\;f\left(r,\phi \right)\;r\;dr\;d\phi }{{\;\int \int \;|\zeta \left(r,\phi \right)|}^{2}\;r\;dr\;d\phi }$$

where 〈·〉 denotes the guided-mode-weighted transverse averaging operator for any position-dependent physical quantity f(r,ϕ). Since the probe field and the generated FWM field are guided modes, the effective contribution of each transverse position is determined by the guided-mode intensity distribution. The near-surface region contributes more strongly because the evanescent guided field is more intense there. Therefore, a guided-mode-weighted average is introduced to convert the local nonlinear response into an effective one-dimensional propagation coefficient. After spatially weighted averaging, the effective coupling coefficients are given by

(19) $${\bar{g}}_{2}=\langle g_{2}\rangle ,\;{\bar{g}}_{4}=\langle g_{4}\rangle ,\;{\bar{f}}_{2}=\langle f_{2}\rangle ,\;{\bar{f}}_{4}=\langle f_{4}\rangle$$

The overbar $\bar{\left(\cdot \right)}$ marks the mode-weighted effective one-dimensional coupling coefficients after transverse averaging. In the subsequent numerical calculations, the fiber cross section is discretized into a computational grid. The local coefficients are calculated at each grid point and then summed with the weighting function Equation (18).

## 3. Results and Discussion

### 3.1. Detuning Characteristics of the Mode-Weighted FWM Response

**Figure 2:**
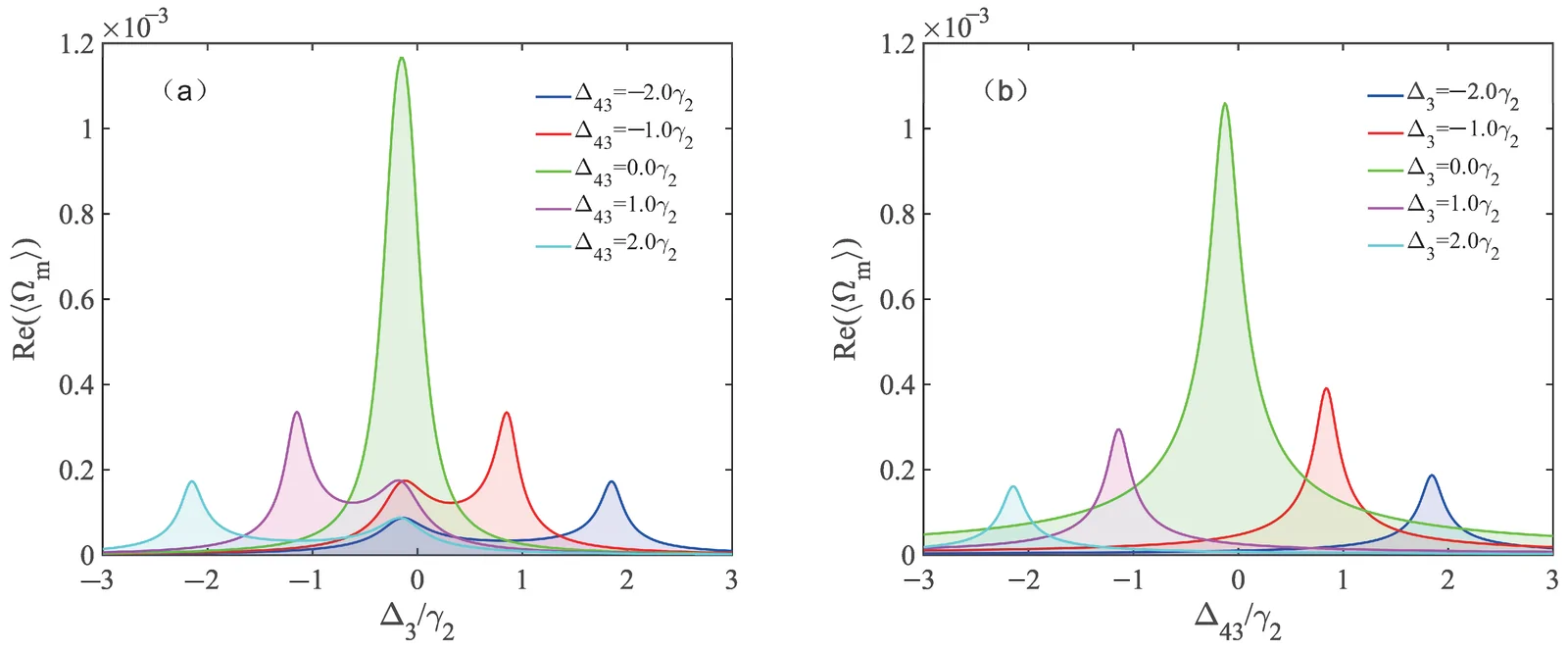
Calculated effective FWM response Re(〈Ω˜m〉) as a function of the detuning parameters. (**a**) Re(〈Ω˜m〉) as a function of the normalized two-photon detuning $\Delta_{3}/\gamma_{2}$ for different values of $\Delta_{43}/\gamma_{2}=-2,-1,0,1,2$. (**b**) Re(〈Ω˜m〉) as a function of the normalized upper-transition detuning $\Delta_{43}/\gamma_{2}$ for different values of $\Delta_{3}/\gamma_{2}=-2,-1,0,1,2$. The parameters are $\Omega_{c}=\gamma_{2}$, $\Omega_{p}^{0}=0.05\gamma_{2}$, $\Omega_{1}=\Omega_{2}=0.1\gamma_{2}$, $\gamma_{3}=0.2\gamma_{2}$, $\gamma_{4}=0.1\gamma_{2}$, $\gamma_{1}=0.001\gamma_{3}$, $\Delta_{2}=0$, and $\omega =0.15\gamma_{2}$.

Based on the theoretical model and numerical solution framework established above, we further investigate how the FWM response in the nanofiber local-coupling system responds to variations in detuning parameters. Unlike conventional free-space models that assume a spatially uniform probe field, the present model incorporates the transverse distribution of the nanofiber-guided probe mode and evaluates the nonlinear response through guided-mode-weighted spatial averaging. According to the coupled-wave equation, the generated FWM field is driven by the source term $g_{4}{\tilde{\Omega }}_{p}$. In the present numerical treatment, the local FWM source response is characterized by $\left|g_{4}\Omega_{p}\left(r,\phi \right)\right|$ and evaluated through guided-mode-weighted spatial averaging. For simplicity, the resulting effective FWM response is denoted by Re(〈Ω˜m〉) in Figure 2. Figure 2a shows the variation in Re(〈Ω˜m〉) with $\Delta_{3}$ under different values of $\Delta_{43}$, while Figure 2b shows its variation with $\Delta_{43}$ under different values of $\Delta_{3}$. These two sets of results indicate that the effective FWM source response exhibits clear resonant behavior with respect to both detuning parameters and that the peak positions shift systematically as the other detuning parameter is varied.

As seen from Figure 2, the weighted FWM response is strongest when the multiphoton resonance condition is satisfied, namely when $\Delta_{3}$ and $\Delta_{43}$ are close to their optimal values. As either detuning deviates from its optimal value, the resonance peak decreases rapidly and the peak position shifts systematically with the other detuning parameter. This indicates that, in the present system, the two detuning conditions do not act independently; instead, they jointly determine the optimal FWM condition through a common multiphoton coherent process [3,32]. Physically, the two-photon detuning controls the efficiency of establishing coherent population and polarization among the lower levels, whereas the upper-transition detuning affects whether the higher-order nonlinear polarization channel can accumulate efficiently [1,32]. When both of them are simultaneously close to resonance, the coherent coupling in the system is established most effectively and linear absorption together with additional loss is suppressed, resulting in a significant enhancement of the generated mixing field [3,32]. When either detuning departs from its optimal value, the multiphoton coherent channel is disrupted and the nonlinear polarization efficiency is reduced. Consequently, the effective FWM response decreases.

Moreover, the peaks in Figure 2 are not fixed at a single detuning value, but shift noticeably as the other detuning parameter changes. This behavior originates from the fact that the effective nonlinear response is governed by the common denominator *S*, in which the detuning terms and the Rabi-frequency terms are coupled together. Therefore, changing $\Delta_{3}$ changes the effective resonance condition for $\Delta_{43}$, and vice versa. In this treatment, the spatial averaging does not represent a simple volume average. Instead, each atomic position contributes according to the local guided-mode intensity, meaning that regions with stronger guided probe intensity contribute more strongly to the effective FWM response. Physically, this reflects the fact that the present FWM process is not determined by a single transition, but instead results from the combined action of multiple transition channels and multiple coherence terms.

The nanofiber local-coupling environment also plays an important role in this process. Because the subwavelength guided mode possesses a small mode volume and a pronounced evanescent-field distribution, the system can sustain a much stronger local electric field under the same input power, thereby increasing the effective atomic Rabi frequencies [15,31]. Since FWM fundamentally relies on high-order nonlinear polarization induced by the coupling among multiple fields [15,31], local-field enhancement can strengthen the FWM response. At the same time, the nonuniform transverse profile of the guided probe field makes the local FWM source response position dependent. The guided probe field is not uniform in the transverse direction, so different spatial regions contribute differently to the effective FWM response. Therefore, the response shown in Figure 2 should be understood as a weighted superposition of local spatial responses rather than a simple average under the uniform-field approximation [15,31]. This mode-weighted source response provides the basis for the subsequent calculation of the FWM conversion efficiency.

### 3.2. Enhancement Characteristics of the Four-Wave-Mixing Efficiency

**Figure 3:**
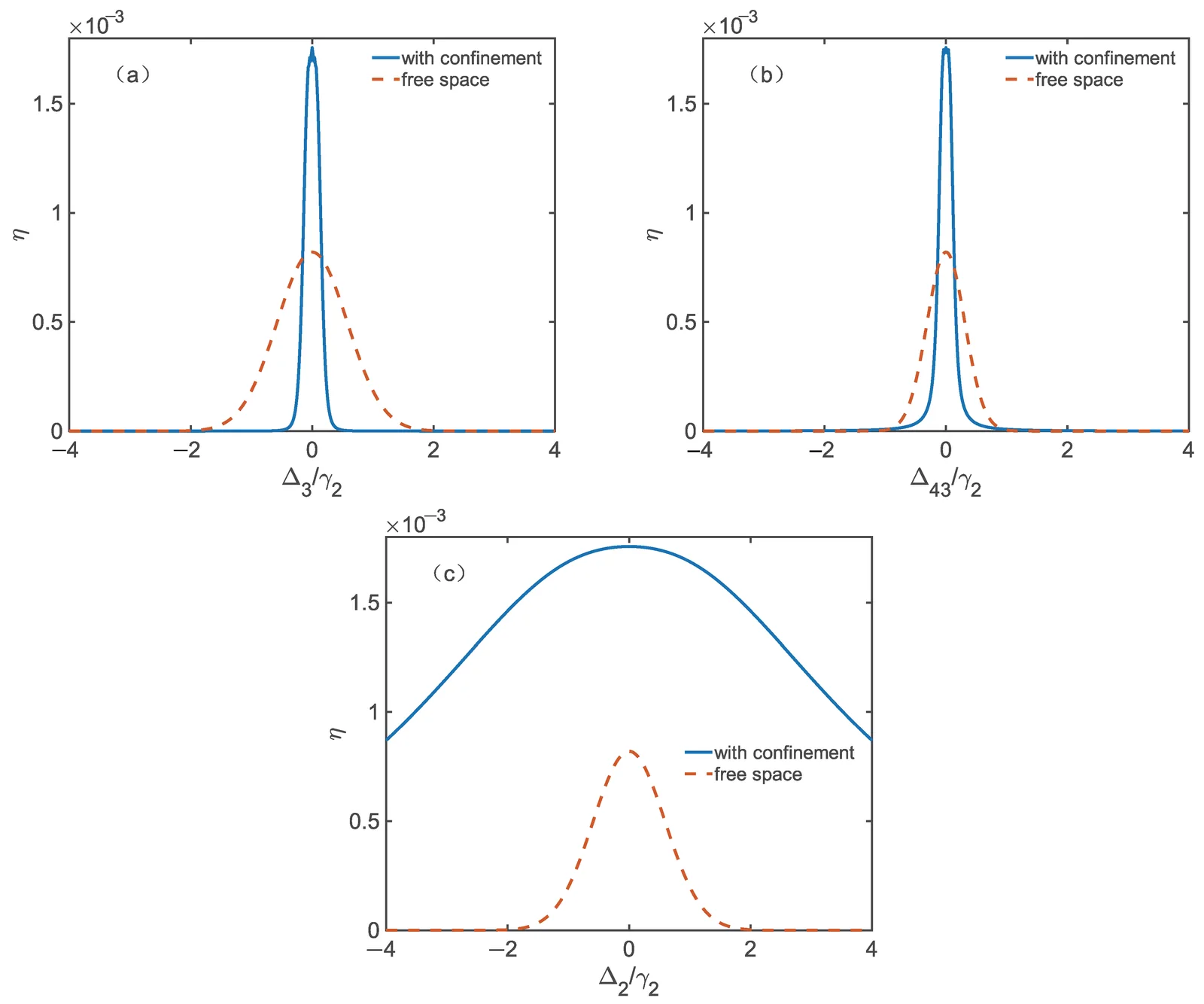
(**a**) Calculated FWM conversion efficiency η as a function of the normalized two-photon detuning $\Delta_{3}/\gamma_{2}$ with $\Delta_{43}=0$ and $\Delta_{2}=0$. (**b**) Calculated FWM conversion efficiency η as a function of the normalized three-photon detuning $\Delta_{43}/\gamma_{2}$ with $\Delta_{3}=0$ and $\Delta_{2}=0$. (**c**) Calculated FWM conversion efficiency η as a function of the normalized pump detuning $\Delta_{2}/\gamma_{2}$ with $\Delta_{3}=0$ and $\Delta_{43}=0$. In each panel, the solid curve represents the result obtained with nanofiber spatial mode confinement, while the dashed curve corresponds to the conventional free-space uniform-field case. The parameters are $\Omega_{c0}=0.5\gamma_{2}$, $\Omega_{p0}=0.02\gamma_{2}$, $\Omega_{10}=\Omega_{20}=0.1\gamma_{2}$, $\gamma_{3}=0.14\gamma_{2}$, $\gamma_{4}=0.015\gamma_{2}$, $\gamma_{1}=0.001\gamma_{3}$, $\kappa_{02}=\gamma_{2}/L$, $\kappa_{04}=\kappa_{02}/100$, L=0.002, and δk=0. The input pump pulse is assumed to have a Gaussian spectrum with pulse width τ=6/G, where $G=\sqrt{\gamma_{2}^{2}+4\Omega_{c0}^{2}}-\gamma_{2}$.

To further quantify the conversion enhancement induced by the nanofiber-confined optical fields, we investigate the dependence of the FWM efficiency η on the key system parameters. To quantify the efficiency enhancement enabled by EIT, we calculate the conversion efficiency η defined in Equation (17). The pump field is taken to be a Gaussian pulse $\Omega_{p}\left(t\right)=\Omega_{p0}\exp\left(-t^{2}/\tau^{2}\right)$, whose pulse width τ satisfies the adiabatic condition τ≥1/Γ, where $\Gamma =\sqrt{\gamma_{2}^{2}+4\Omega_{c}^{2}}-\gamma_{2}$ is the EIT half-width at half maximum [3,32]. The results are shown in Figure 3. The FWM efficiency exhibits pronounced sharp peaks near the resonance regions of $\Delta_{3}$ and $\Delta_{43}$, whereas its dependence on the single-photon probe detuning $\Delta_{2}$ shows a broader and more tolerant peak profile. This indicates that different detuning parameters play different roles in the FWM process: $\Delta_{3}$ and $\Delta_{43}$ mainly determine the multiphoton coherent resonance condition, while $\Delta_{2}$ mainly tunes the overall excitation and propagation response.

As can be seen from Figure 3a,b, the FWM efficiency reaches its maximum when the two-photon and three-photon detunings are close to zero, and decays rapidly when they move away from resonance. Such sharp peak structures indicate that the conversion efficiency is highly selective with respect to the resonance-matching condition. Comparing the solid curves (with spatial confinement) and dashed curves (free-space), one can observe that spatial confinement substantially elevates the peak efficiency in both subplots. For the two-photon detuning $\Delta_{3}/\gamma_{2}$ in Figure 3a, the resonant peak becomes notably narrower under confinement, making the system more sensitive to detuning deviations; by contrast, for the three-photon detuning $\Delta_{43}/\gamma_{2}$ in Figure 3b, the spectral width of the resonance barely changes despite the enhanced peak amplitude. The essential reason is that the two-photon and three-photon detunings directly determine whether the key coherent channels in the FWM process can be opened efficiently. When the system is tuned close to both the two-photon resonance and the three-photon resonance, the coherent superposition associated with the nonlinear polarization process becomes most complete [1,32,33]. Under this condition, absorption loss is strongly suppressed by the EIT mechanism. Once the detuning parameters move away from the optimal region, the coherent coupling is quickly destroyed, leading to a rapid decrease in efficiency.

In contrast, Figure 3c shows that the variation in FWM efficiency with pump detuning $\Delta_{2}$ presents a broader single-peak structure whose maximum is likewise located near zero. A distinct feature under spatial confinement is that not only the peak efficiency is improved, but the overall spectral bandwidth is further broadened relative to the free-space case, which yields better tolerance against pump-frequency drift. This means that although the pump detuning also affects the FWM efficiency, it does not need to be precisely zero, unlike the two-photon and three-photon detunings, in order to activate the resonance condition. Instead, it influences the FWM efficiency mainly by adjusting the overall excitation strength of the system. For this reason, the η-versus-$\Delta_{2}$ curve is smoother and more tolerant to parameter deviations. In other words, once the resonance-matching condition is maintained, the pump field can still continuously tune the system-driving strength over a certain range without immediately destroying the condition for high-efficiency conversion.

Furthermore, the efficiency distribution shown in Figure 3 also reflects the advantage of the nanofiber local-coupling system over the conventional free-space configuration [1,15]. Because the nanofiber guided mode has a much stronger local field-confinement capability, atoms near the fiber surface experience a larger effective field strength and are therefore more likely to establish a strong nonlinear polarization response under the same input conditions. Consequently, the high-efficiency peaks in Figure 3 originate not only from the EIT-induced suppression of absorption in the usual sense, but also from the mode-localization enhancement provided by the nanofiber [15,32]. It is worth noting that spatial confinement exerts differentiated modifications on spectral profiles for different detuning channels: The spectral response exhibits distinct dependence on the detuning parameters. Compared with the uniform-field reference, the resonance corresponding to the two-photon detuning $\Delta_{3}$ becomes narrower, while the bandwidth for the upper-transition detuning $\Delta_{43}$ remains largely unchanged. In contrast, the range of probe detuning $\Delta_{2}$ over which appreciable conversion is obtained becomes broader, and broadens the usable bandwidth for pump detuning. Therefore, compared with a homogeneous-field approximation, the nanofiber-guided-mode model predicts a more realistic efficiency landscape determined by both local field enhancement and spatially varying atom–field interaction. From an application point of view, the system should prioritize accurate matching of the two-photon and three-photon resonance conditions, while the pump detuning can be optimized within a relatively broad range so as to balance high efficiency and experimental robustness.

### 3.3. Multi-Parameter Optimization and Search for the Maximum Mixing Efficiency

In the previous subsection, we analyzed the response of the FWM output amplitude and the conversion efficiency to variations in detuning parameters. The results showed that the FWM efficiency depends nonlinearly on the two-photon detuning, the three-photon detuning, and the pump detuning, and that sharp peaks appear near the resonant conditions. However, because the present nanofiber local-coupling system involves spatially dependent nonlinear coupling, multilevel coherence, and several coupled detuning parameters, the efficiency landscape becomes highly nonlinear, making it difficult to determine the global optimum operating point through single-parameter scans alone. It is therefore necessary to carry out a joint optimization over multiple parameters within physically meaningful ranges in order to identify the parameter combination that maximizes the FWM efficiency.

In the present system, the main tunable parameters affecting the FWM efficiency include the two-photon detuning $\Delta_{3}$, the three-photon detuning $\Delta_{43}$, the pump detuning $\Delta_{2}$, the coupling-field Rabi frequency, the mode-volume distribution, and the atomic decay rates. Among them, the detuning parameters influence the establishment of nonlinear polarization most directly. Therefore, in this work, $\Delta_{3}$, $\Delta_{43}$, and $\Delta_{2}$ are selected as the principal optimization variables, while the remaining physical parameters are fixed according to the experimentally reasonable values and theoretical model conditions:

(20) $$-4\gamma_{2}\leq \Delta_{3}\leq 4\gamma_{2},\;-4\gamma_{2}\leq \Delta_{43}\leq 4\gamma_{2},\;-4\gamma_{2}\leq \Delta_{2}\leq 4\gamma_{2}$$

The mixing efficiency η is taken as the optimization objective function. The efficiency function is jointly determined by the multi-level coupling equation and the propagation equation, and its expression contains multiple complex denominator terms and phase-matching terms, presenting a highly nonlinear characteristic. Moreover, there may be multiple local extreme points in the parameter space. Therefore, the Particle Swarm Optimization (PSO) algorithm is employed to perform a global search in the multidimensional parameter space. Compared with independent parameter scanning, PSO can simultaneously consider the coupling among different detuning parameters and identify the parameter combination that maximizes the FWM efficiency [34,35]. PSO is a typical meta-heuristic optimization method, featuring simple implementation, fast convergence speed, and advantages in complex nonlinear search spaces. It has been widely applied in complex nonlinear optical parameter optimization problems [34,35,36]. By using the mixing efficiency function as the fitness function and continuously updating the particle positions within the given parameter range, the maximum efficiency can be gradually approximated.

In the numerical calculations, each iteration requires solving the coupled propagation equations and carrying out weighted averaging over both the frequency distribution and the transverse mode distribution, so as to obtain the total FWM efficiency corresponding to the given parameter set. The PSO search yields the highest conversion efficiency obtained within the specified parameter range, together with the corresponding optimal parameter set, and the results are summarized in Table 1:

Table 1 shows that, after the multi-parameter joint optimization, the maximum FWM efficiency of the system reaches $\eta =1.85\times 10^{-3}$, which is significantly higher than the values obtained from single-parameter scans. This demonstrates that the optimal FWM efficiency is determined by the cooperative optimization of multiple parameters rather than by a single resonance condition. The optimized point represents the balance among multiphoton resonance, nonlinear polarization generation, and propagation efficiency.

**Figure 4:**
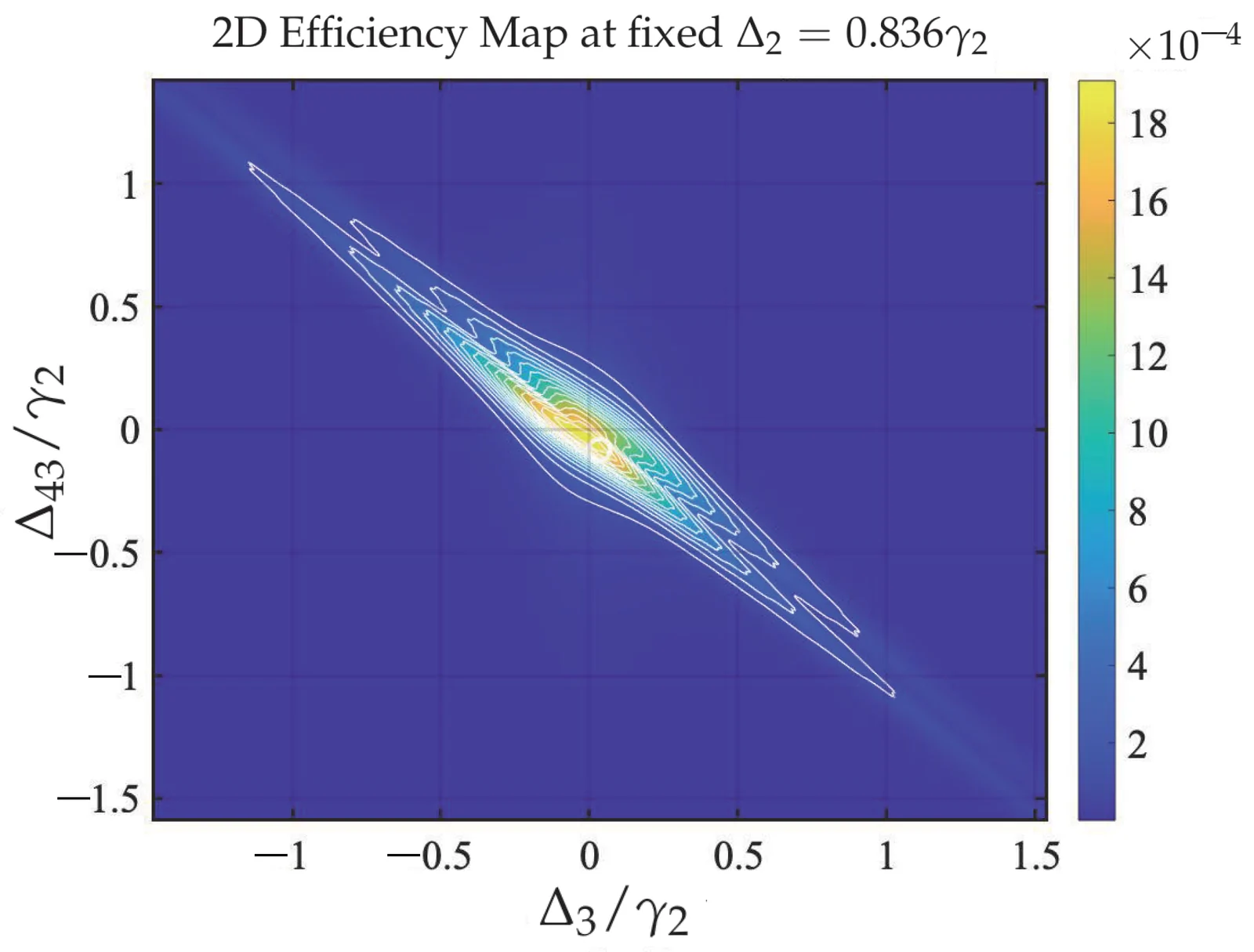
Two-dimensional map of the FWM conversion efficiency η in the $\left(\Delta_{3}/\gamma_{2},\Delta_{43}/\gamma_{2}\right)$ parameter plane at a fixed pump detuning $\Delta_{2}=0.836\gamma_{2}$. The color scale represents the magnitude of the conversion efficiency. The white circle indicates the optimal operating point, at which the conversion efficiency reaches its maximum value.

To further analyze the efficiency distribution near the optimal point, a two-dimensional scan over $\Delta_{3}$ and $\Delta_{43}$ is performed in the vicinity of the optimized parameters, which yields the efficiency map shown in Figure 4. It can be seen that the efficiency forms an obvious oblique high-value band in parameter space, and that the maximum lies near the center of this band, in agreement with the optimum obtained from particle swarm optimization.

This oblique distribution indicates that the high-efficiency region is not simply located at $\Delta_{3}=0$ and $\Delta_{43}=0$. Such a tilted high-efficiency region further confirms that the mode-weighted nonlinear interaction and atomic coherence pathways jointly determine the optimal operating condition. It reveals strong coupling among the different transition channels inside the system, so that a change in one detuning redefines the optimal value of the other and causes the best operating condition to move along a specific direction. This phenomenon further confirms that the FWM process in the present system is governed by the cooperative action of multiple resonant pathways rather than by an isolated single transition.

In summary, multi-parameter joint optimization can identify the optimal operating region for the FWM efficiency and further demonstrate the clear advantage of the nanofiber local-coupling system in achieving highly efficient nonlinear frequency conversion. Compared with simpler three- or four-level atomic configurations, the five-level scheme offers greater flexibility in tailoring the nonlinear optical response through its additional driven transitions. This increased tunability, however, does not necessarily translate into higher conversion efficiency. The implementation requires more precise control of the driving fields, while the additional excitation pathways may complicate population management and introduce further sources of decoherence. Moreover, since the present study does not include a direct comparison with optimized three- or four-level systems, no quantitative performance advantage over these simpler configurations can be established. The main potential benefit of the five-level arrangement therefore lies in its additional control over the nonlinear response, whose practical value must be weighed against the increased demands of experimental implementation.

## 4. Conclusions

We have examined EIT-assisted four-wave mixing (FWM) in a five-level atom–nanofiber system. The transverse variation in the guided field was accounted for using a guided-mode-weighted mean-field approximation to obtain effective one-dimensional propagation coefficients. Under the adopted normalization, the calculated peak conversion efficiency exceeds that of the selected uniform-field reference for the parameters studied. The spectral changes are detuning dependent: the two-photon resonance associated with $\Delta_{3}$ narrows, while the bandwidth associated with the upper-transition detuning $\Delta_{43}$ remains nearly unchanged. Appreciable conversion also persists over a wider range of probe detunings $\Delta_{2}$. A particle swarm optimization (PSO) search identifies a best-found conversion efficiency of $1.85\times 10^{-3}$ within the prescribed parameter bounds. The two-dimensional efficiency map contains a tilted high-efficiency band, indicating that favorable operating conditions depend on correlated detunings rather than separately tuned resonances. Together, these findings show how the transverse guided-mode profile and the interplay among detunings shape the calculated FWM response. Quantitative comparison with experiments will require transition-specific atomic parameters, wavelength-dependent guided-mode profiles, and a more complete treatment of experimental broadening effects.

## Figures and Tables

**Table 1:** Optimized parameters and maximum FWM efficiency.

Parameter	Value (Hz)	Normalized Value
$\Delta_{3}$	$-5.70\times 10^{4}$	$-0.057\;\gamma_{2}$
$\Delta_{43}$	$6.58\times 10^{3}$	$0.0066\;\gamma_{2}$
$\Delta_{2}$	$8.36\times 10^{5}$	$0.836\;\gamma_{2}$
$\Delta_{4}$	$-5.04\times 10^{4}$	$-0.050\;\gamma_{2}$
Efficiency η	$1.85\times 10^{-3}$	

## Data Availability

The data and numerical codes supporting the findings of this study are available from the corresponding author upon reasonable request.

## Abbreviations

The following abbreviations are used in this manuscript:

FWM	Four-wave mixing
EIT	Electromagnetically induced transparency
PSO	Particle swarm optimization
RWA	Rotating-wave approximation
HE_11_	Fundamental hybrid guided mode
